# Modeling *Neisseria meningitidis *metabolism: from genome to metabolic fluxes

**DOI:** 10.1186/gb-2007-8-7-r136

**Published:** 2007-07-06

**Authors:** Gino JE Baart, Bert Zomer, Alex de Haan, Leo A van der Pol, E Coen Beuvery, Johannes Tramper, Dirk E Martens

**Affiliations:** 1 Unit Research & Development, Netherlands Vaccine Institute (NVI), PO Box 457, 3720 AL Bilthoven, The Netherlands; 2 PAT Consultancy, Kerkstraat 66, 4132 BG Vianen, The Netherlands; 3 Food and Bioprocess Engineering Group, Wageningen University, PO Box 8129, 6700 EV Wageningen, The Netherlands

## Abstract

**Background:**

*Neisseria meningitidis *is a human pathogen that can infect diverse sites within the human host. The major diseases caused by *N. meningitidis *are responsible for death and disability, especially in young infants. In general, most of the recent work on *N. meningitidis *focuses on potential antigens and their functions, immunogenicity, and pathogenicity mechanisms. Very little work has been carried out on *Neisseria *primary metabolism over the past 25 years.

**Results:**

Using the genomic database of *N. meningitidis *serogroup B together with biochemical and physiological information in the literature we constructed a genome-scale flux model for the primary metabolism of *N. meningitidis*. The validity of a simplified metabolic network derived from the genome-scale metabolic network was checked using flux-balance analysis in chemostat cultures. Several useful predictions were obtained from *in silico *experiments, including substrate preference. A minimal medium for growth of *N. meningitidis *was designed and tested succesfully in batch and chemostat cultures.

**Conclusion:**

The verified metabolic model describes the primary metabolism of *N. meningitidis *in a chemostat in steady state. The genome-scale model is valuable because it offers a framework to study *N. meningitidis *metabolism as a whole, or certain aspects of it, and it can also be used for the purpose of vaccine process development (for example, the design of growth media). The flux distribution of the main metabolic pathways (that is, the pentose phosphate pathway and the Entner-Douderoff pathway) indicates that the major part of pyruvate (69%) is synthesized through the ED-cleavage, a finding that is in good agreement with literature.

## Abstract 2

A genome-scale flux model for primary metabolism of *Neisseria meningitidis *was constructed; a minimal medium for growth of *N. meningitidis *was designed using the model and tested successfully in batch and chemostat cultures.

## Background

*Neisseria meningitidis *is a human pathogen that can infect diverse sites within the human host. The major diseases caused by *N. meningitidis *- meningitis and meningococcal septicemia - are responsible for death and disability, especially in young infants. There are different pathogenic *N. meningitidis *isolates, of which serogroups B and C cause the majority of infections in industrialized countries, whereas strains of group A and C dominate in less developed countries [1]. Some disease control has been achieved by vaccination with polysaccharide vaccines. Effective conjugate vaccines against group C organisms have been licensed in the United Kingdom and other countries [2]. At present there is no vaccine available against group B organisms, which are the predominant cause of meningococcal disease in developed countries [3]. Development of a safe and effective vaccine based on the serogroup B capsular polysaccharide is complicated because of the existence of identical structures in the human host [4]. This results in poor immunogenicity and the risk of inducing autoimmunity [5].

Current strategies for developing a vaccine to prevent disease caused by serogroup B meningococci include outer membrane protein- and lipopolysaccharide-based approaches [3]. In addition, the systematic search of genomic information, termed 'reverse vaccinology', has been used to identify novel protein antigens [6-10]. Genomic-information-based analysis of pathogens has dramatically changed the scope for developing improved and novel vaccines by increasing the speed of target identification in comparison with conventional approaches [11].

The outer membrane protein PorA has been identified as a major inducer of, and target for, serum bactericidal antibodies and is expressed by almost all meningococci, which pinpoints PorA as a promising vaccine candidate [12]. However, PorA appears to be heterogeneous, which will mean the development of a multivalent vaccine in which various porA subtypes are present in order to induce sufficient protection. Although various approaches can be used in the development of a multivalent vaccine, the use of genetically engineered strains expressing more than one PorA subtype to overcome the problem of heterogeneity seems promising [13]. At the Netherlands Vaccine Institute (NVI), a vaccine against serogroup B meningococci is currently being developed. It is based on different PorA subtypes contained in outer membrane vesicles (OMVs). An important aspect of this development trajectory is the process development of the cultivation step, which includes, for example, the design of a culture medium. Genome-scale constraints-based metabolic models are useful tools for this.

**Figure 1:**
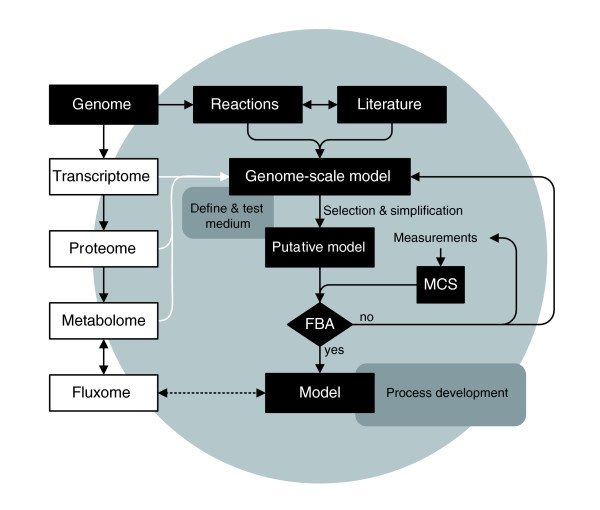
Schematic representation of model construction. The genome can be classified as the first-level database. holding the potential functions of an organism. The transcriptome can be classified as the second-level database of functions describing the actual expression of genes, and the proteome can be classified as the third-level database of functions describing the actual expressed proteins. The metabolome (and fluxome) can be classified as the fourth-level database holding the complete collection of metabolites and reactions in which the metabolites participate. The metabolome, and to a lesser extent the proteome, determine the functionality of the cell [146]. In principle, all databases can be used as source of input for construction (or extension) of a genome-scale model (white arrows). In our study, information provided by the genome and the literature was used for model construction (black boxes). A minimal medium for growth was derived from the genome-scale model (upper gray box). The genome-scale model was simplified as descibed in the text, resulting in the 'putative model'. The measured specific metabolic rates and the corresponding measurement variances used in flux balance analysis (FBA) were calculated using Monte Carlo Simulation (MCS) with the measured experimental data and their standard deviation as input. The final model, verified by FBA, can be used for process development purposes (for example, optimization of growth medium, lower gray box). Subsequently, the model can be extended to the desired informative level using all available sources of information (light gray circle).

In general, most of the recent work on *N. meningitidis*, whether based on genomic information or not, focuses on potential antigens and their functions, on immunogenicity, and on pathogenicity mechanisms. Very little work has been carried out on *Neisseria *primary metabolism over the past 25 years. However, the information provided by the genome can also be used to obtain information on the metabolic capabilities of the organism. This is done by screening the genome for open reading frames (ORFs) that code for enzymes present in the primary metabolism, yielding a genome-scale metabolic network. Such a network may still contain gaps due to the incomplete or incorrect annotation of the genome. Using biochemical literature, transcriptome data or by direct measurements, the presence of missing enzymatic reactions may be proved and the network can be completed. Often, such a complete model contains underdetermined parts due to the presence of parallel or cyclic pathways. This means that for certain parts of the network the flux values cannot be determined. In order to narrow down the number of possible solutions for these parts, constraints can be set on certain enzymatic reactions on the basis of biochemical and thermodynamic information found in the literature or determined experimentally. A schematic diagram of how the genome-scale flux model was constructed and verified using flux balance analysis (FBA) is shown in Figure 1.

Genome-scale constraints-based metabolic models have been built for several organisms, as summarized by Palsson and colleagues [14]. They can be used to analyze culture data, to get a better understanding of cellular metabolism, to develop metabolic engineering stategies [15-17], design of media and processes [17-19] and even for online control of the process [20]. Knowledge about metabolism, as contained in these models, is very useful for the development of an efficient cultivation process. Notably, product quality and quantity are primarily determined in the cultivation process.

The aim of this study was to construct a metabolic model of serogroup B *N. meningitidis *(MenB) based on genome annotation and the biochemical literature for effective process development purposes. The model was verified experimentally using flux-balance analysis (FBA) for steady-state chemostat data.

## Results and discussion

### Construction of the genome-scale metabolic model

**Figure 2:**
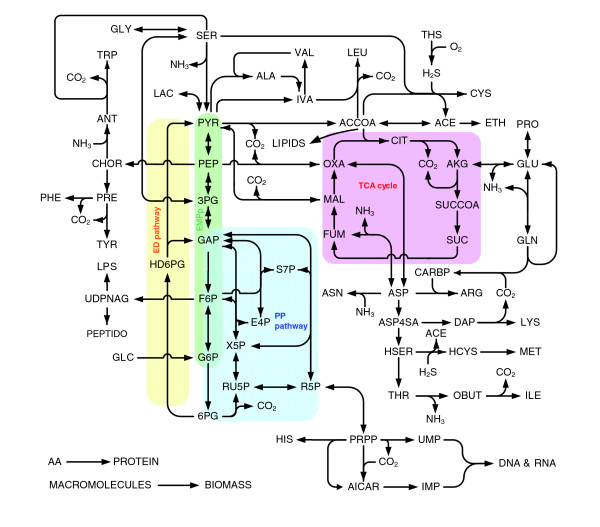
Simplified metabolic model of *N. meningitidis*. As described in the text, the simplified model was obtained by simplification of the genome-scale model. For ease of understanding, only the main pathways were admitted into the diagram illustrated here. A complete overview of the model including a list of all abbreviations used is in Additional data file 1.

The available genome sequence of MenB [21] and its annotation in the Kyoto Encyclopedia of Genes and Genomes (KEGG) database [22] was taken as starting point for model construction. As described by Heinemann and co-workers [23], the KEGG database was corrected for obvious errors [24] and complemented using the database of The Institute for Genome Reearch (TIGR) [25], which is based on the same sequence data, but runs a different annotation methodology, and the BioCyc database [26]. These databases, along with biochemical information found in the literature, provided the information needed to construct the genome-scale metabolic model. The genome-scale model was next simplified by lumping successive reactions together and removing dead ends. This led to the simplified model shown in Figure 2. The complete reaction database along with the genes involved, enzyme numbers and metabolites is in Additional data file 1. From this reaction database, parts that describe the main primary pathways were selected and cross-checked with the literature.

#### Glucose metabolism

According to the genomic information for MenB, glucose can be completely catabolized through the Entner-Douderoff pathway (ED) and the pentose phosphate pathway (PP). The Embden-Meyerhof-Parnas glycolytic pathway (EMP) is not functional, because the gene for phosphofructokinase (EC 2.7.1.11) is not present. Studies on the utilization of glucose in *N. meningitidis *[27-33] confirm the presence of enzymes related to the EMP, ED and PP pathways. However, it was found that the EMP pathway does not contribute to pyruvate synthesis, indicating that, in accordance with the missing phosphofructokinase gene, this pathway is not functional. On the basis of genomic information, glyceraldehyde phosphate and fructose-6-phosphate, which are formed in the PP pathway, can be recycled to glucose-6-phosphate. This indicates that in theory glucose can be completely oxidized in the PP pathway to CO_2_, forming six NADPH. In practice, it was found that glucose is mainly catabolized by the ED pathway and to a lesser extent by the PP pathway [32]. On the basis of ^14^C studies, Jyssum [32] roughly calculated that the ED cleavage always synthesizes the major part of pyruvate (67-87 %) and the PP pathway accounts for the remaining part. Morse and co-workers [34] found similar results for *Neisseria gonnorhoeae*, which is biochemically similar to *N. meningitidis *[35].

#### The tricarboxylic acid cycle

All the genes for the tricarboxylic acid cycle (TCA cycle, citric acid cycle) enzymes are present in the MenB genome, except for the gene for malate dehydrogenase. To establish oxidation of malate to oxaloacetate, the MenB genome suggests the FAD-dependent malate:quinone oxidoreductase (NMB2069). Studies of the TCA cycle and related reactions [35-42] in *N. meningitidis *and *N. gonorrhoeae *indeed confirm the presence of all citric-acid-cycle enzymes except malate dehydrogenase. Although activity of the FAD-dependent malate:quinone oxidoreductase has not been measured, its presence is plausible as indicated by an operational TCA cycle. The TCA cycle, ED pathway, PP pathway and the dysfunctional EMP pathway are shown schematically in Figure 2.

#### Anaplerotic reactions

In the MenB genome, a gene for phosphoenolpyruvate carboxylase (NMB2061) is present. Studies on phosphoenolpyruvate carboxylase activity [35,43,44] confirm the presence of a specific irreversible phosphoenolpyruvate carboxylating activity, EC 4.1.1.31. Other phosphoenolpyruvate-carboxylating enzymes have not been annotated in the MenB genome. In addition, the gene for malic enzyme, EC 1.1.1.38 (NMB0671), is present in the genome. On the basis of genomic information, *N. meningitidis *does not posses a functional glyoxylic acid cycle, as the genes for isocitrate lyase (EC 4.1.3.1) and malate synthase (EC 2.3.3.9) are not present. This was confirmed by Holten [45] and Leighton [35], who both did not detect these enzymes in cell-free extracts.

#### Metabolism of lactate, acetate, glutamate and carbon dioxide

The first work on the growth requirements of *Neisseria *in a chemically defined environment was published in 1942 [46] and provided the basis for many later metabolic studies. Growth of *N. meningitidis *requires glucose, pyruvate, or lactate as sole carbon source, and during cultivation on any of these carbon sources, secretion of acetate into the medium occurs [47,48]. In addition, a certain environmental CO_2 _tension was required to initiate growth [49]. *In silico *simulation of biomass growth using the metabolic model showed that when bacteria are grown on glucose and the oxidation capacity is limiting, acetate secretion occurs. Limitations in oxidation capacity may be due to limitations in metabolism or limitations in oxygen supply to the culture. *In silico *simulation of limited oxygen supply was added as an example to Additional data file 1.

Studies on lactate utilization [50] showed that L-lactate can be utilized by different meningococcal lactate dehydrogenases (LDH). In the MenB genome, an LDH gene (NMB1377) specific for L-lactate (EC1.1.2.3, EC 1.1.1.27) has been annotated. The predicted amino-acid sequence of this gene, *lldA*, is homologous to that of the *Escherichia coli lldD *gene (43% similarity) and to other prokaryotic and eukaryotic flavin mononucleotide-containing enzymes that catalyze the oxidation of L-lactate. However, in *E. coli *the corresponding *lldD *gene is part of an L-lactate-regulated operon also containing genes for *lldP *(permease) and *lldR *(regulatory), whereas the meningococcal L-LDH gene does not appear to be part of an operon [50]. A meningococcal *lld *mutant had reduced L-LDH activity, but was still able to grow on L-lactate, indicating that a second L-LDH must exist [50]. At present, no additional genes have been annotated.

Two LDH genes specific for D-lactate (EC 1.1.1.28) have been annotated in the MenB genome. These genes, NMB0997 and NMB1685, are homologous to the *E. coli dld *and *ldhA *genes, respectively (71% and 66% similarity). In agreement with this, an NAD-dependent D-LDH activity was identified by Erwin and Gotschlich [51]. *ldhA *is associated with fermentative processes [52]. L-lactate is an established and important intermediate in mammalian metabolism. It is less clear whether D-lactate also originates from mammalian metabolism. D-lactate can be produced as a byproduct of glucose metabolism by some lactic acid bacteria as well as by *E. coli*, so it may be available on the mucosal surfaces that pathogenic *Neisseria *colonize. De Vrese and co-workers [53] showed that after human consumption of food containing DL-lactic acid (such as yoghurt), significant levels of both L- and D-lactic acid were present in the blood. Although both L- and D-lactic acids were metabolized rapidly, the availability of D-lactate in humans might explain the presence of specific D-lactate dehydrogenases in *N. meningitidis*. Erwin and Gotschlich [51] showed that *N. meningitidis *was able to grow on L-lactate at least as well as on glucose, but more recent work of Leighton [35] contradicts this. He found that growth on L-lactate gave a lower biomass yield and suggested that the additional ATPs produced during glucose catabolism to pyruvate, which are not formed when growing on lactate, accounted for this observation. In addition, the C_5 _and C_6 _carbohydrates required for bioynthesis of macromolecules are synthesized using the gluconeogenesis pathway when grown on lactate, which also requires additional ATP. *In silico *simulation of biomass growth using the metabolic model confirmed the observation of Leighton [35] and predicted a 10% higher yield of biomass on glucose.

According to genomic information, acetate is synthesized via phosphate acetyltransferase, EC 2.3.1.8 (NMB0631) and acetate kinase, EC 2.7.2.1 (NMB0435, NMB1518) or acetate-CoA ligase, EC 6.2.1.1 (NMB1555). The presence of phosphate acetyltransferase and acetate kinase was confirmed by activity measurements [35]. The hypothesis that acetate is synthesized from pyruvate [38] via cytochrome-linked pyruvate dehydrogenase (EC 1.2.2.2) might be incorrect, as the required gene is not annotated. Studies on the catabolism of pyruvate and acetate [38] showed that acetate can be oxidized, but only when glutamate is present, indicating that acetate can be oxidized under specific growth conditions. Because the glyoxylate cycle is not present, C_2 _compounds cannot be converted to C_4 _compounds, which explains the requirement for glutamate in this case. *In silico *simulation of biomass growth using the metabolic model confirmed that acetate can be oxidized in the presence of glutamate. The result of this *in silico *experiment can be found in Additional data file 1. Ethanol is synthesized from acetaldehyde using alcohol dehydrogenase, EC 1.1.1.1 (NMB0546). Remarkably, no gene(s) involved in the biosynthesis of acetaldehyde are found in the MenB genome. Because ethanol was measured in the culture supernatant, a biosynthetic pathway to ethanol must be present. Hence, aldehyde dehydrogenase (EC 1.2.1.3) was assumed to be present in the metabolic model to complete the biosynthetic pathway to ethanol.

Both Frantz [46] and Grossowics [48] described glutamate as a requirement in their growth media for meningococci, but Jyssum [54] showed that ammonium can serve as sole nitrogen source after meningococcal adaption to glutamate-free medium. The genes encoding NAD-specific glutamate dehydrogenase (NMB1476) and NADP-specific glutamate dehydrogenase (NMB1710) are present in the MenB genome as well as genes for several aminotransferases. The presence of NAD-dependent glutamate dehydrogenase was demonstrated [54] and additional studies of meningococcal transaminase activity also revealed the presence of transamination to 2-oxoglutarate from a number of amino acid donors. Holten and Jyssum [55] also found NADP-linked glutamate dehydrogenase activity. They found that NAD-linked glutamate dehydrogenase was most active in glutamate-containing media, whereas the NADP-linked enzyme dominated in absence of glutamate [55]. They observed that the NAD-linked enzyme mainly converts glutamate to 2-oxoglutarate and ammonia (catabolism), whereas the NADP-linked enzyme is responsible for the reverse reaction (anabolism). Because in our study glutamate-free medium was used, only the NADP-linked glutamate dehydrogenase was admitted in the metabolic model.

To initiate growth, a certain environmental CO_2 _tension is required [49]. This finding is most probably associated with the high CO_2 _concentration present in the nasopharynx. In more recent studies, additional CO_2 _tension is only used when bacteria are grown on solid media and is omitted in liquid cultures. It seems plausible that the CO_2 _tension is only important in glutamate-free media, where phosphoenolpyruvate carboxylase (NMB2061) must be an important link to the citric acid cycle, whereas this is normally fed by glutamate. This hypothesis is supported by results obtained by Holten [38] and in an earlier study by Jyssum and Jyssum [56], who studied the effect of KHCO_3 _on endogenous phosphorylation.

#### Amino-acid metabolism

All genes involved in amino-acid biosynthesis are present in the MenB genome except the genes coding for alanine transaminase, alanine dehydrogenase, and phosphoglycerate dehydrogenase, which is part of the biosynthetic pathway to serine. For the synthesis of alanine the MenB genome suggests the gene (NMB1823) encoding valine-pyruvate aminotransferase (EC 2.6.1.66). To complete the biosynthesis of serine, phosphoglycerate dehydrogenase was assumed to be present. In 1989, the physiology and metabolism of *N. gonorrhoeae *and *N. meningitidis *was reviewed by Chen and co-workers [57], with emphasis on selected areas that have implications for pathogenesis. The main focus of this work was iron metabolism, and amino-acid metabolism was touched on only briefly for *N. gonorrhoeae*. Catlin [58] investigated growth requirements for various *Neisseria *species, pointing out the additional need for glutamate, arginine, glycine, serine, and cysteine for some *N. meningitidis *strains. However, amino-acid-free growth medium was used earlier [54], indicating that all biochemical pathways for amino-acid synthesis in *N. meningitidis *are available, as supported by the genomic data. This is confirmed by Leighton [35], who measured enrichments of all individual amino-acid carbon after growth on 2-^13^C- and 3-^13^C- labeled pyruvate.

#### Oxidative phosphorylation

MenB genome sequence information indicates the presence of respiratory complexes I, II and III, suggesting that electrons enter the respiratory chain through NADH dehydrogenase (EC 1.6.5.3) or succinate dehydrogenase (EC 1.3.99.1) and are transferred to the cytochrome *bc*_1 _complex through ubiquinone (EC 1.10.2.2). Oxygen is utilized by cytochrome *cbb*_3 _oxidase (EC 1.9.3.1), which is the only respiratory oxidase encoded by the MenB genome. The *cbb*_3_-type oxidases are usually found in proteobacteria that express these oxidases in response to microaerobic conditions to permit the colonization of oxygen-limited environments. Thus *cbb3*-type oxidases may be an important determinant of pathogenicity for MenB [59]. *N. meningitidis *fails to grow under strictly anaerobic conditions. Under oxygen limitation the bacterium expresses a denitrification pathway. This reduction of nitrite to nitric oxide, via nitrite reductase, EC 1.7.2.1 (NMB1623), is regulated by oxygen depletion and nitrite availability [60,61]. Thus, under microaerobic conditions nitrite can replace oxygen as an alternative respiratory substrate in *N. meningitidis*. Because our experiments were not carried out under microaerobic conditions, nitrite was not added to the growth medium, and the denitrification pathway was omitted from the simplified model.

#### Sulfur metabolism

Frantz [46] and Grossowicz [48] described that reduced sulfur in the form of cysteine, cystine, or thiosulfate was required for growth. Catlin [58] showed that some strains of meningococci have an absolute requirement for cysteine (or cystine). Jyssum [54] showed that after adaptation these sulfur sources could be replaced by sulfate. This was confirmed by Port and co-workers [62], who showed that numerous sulfur sources could be used as alternatives to cysteine. DeVoe and co-workers [63] identified thiosulfate reductase activity in *N. meningitidis *serogroup B, but no gene specifically encoding thiosulfate reductase has been annotated in the MenB genome. A wide range of sulfur-acquisition routes is available in *N. meningitidis*. Genes encoding sulfate adenylyltransferase (EC 2.7.7.4), phosphoadenosine phosphosulfate reductase (EC 1.8.4.8), and sulfite reductase (EC 1.8.1.2) are present in the MenB genome. On the basis of this information, both thiosulfate and sulfate were selected as sulfur sources in the growth medium for the production of cysteine and other sulfur-containing compounds.

**Figure 3:**
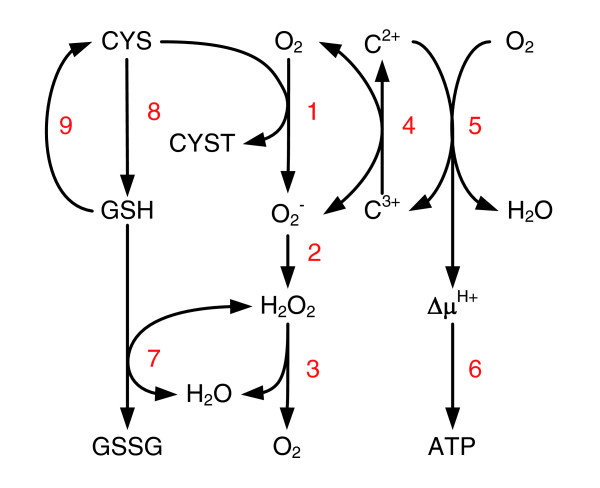
Oxidation of cysteine to cystine. Cysteine (CYS) is oxidized to cystine (CYST), forming reactive oxygen O_2_^- ^(step 1), which can reduce cytochrome *c *(step 4). The electron is used by cytochrome *cbb*_3_, which reduces oxygen to water and causes the concomitant generation of a protonmotive force, Δμ^H+ ^(step 5). The protonmotive force is in turn used to form ATP (step 6). Step 2 involves the formation of hydrogen peroxide (H_2_O_2_) from O_2_^- ^by superoxide dismutase followed by catalase to regenerate oxygen (step 3). Cysteine can be converted to glutathione (GSH), via glutamate-cysteine ligase and glutathione synthetase (step 8). In turn, GSH can be converted to cysteine via gamma-glutamyltranspeptidase and aminopeptidase N (step 9), yielding a functional γ-glutamyl cycle. GSH can be oxidized to glutathione disulfide (GSSG), by glutathione peroxidase (step 7).

Cysteine can be converted to the thiol glutathione (GSH) via glutamate-cysteine ligase, EC 6.3.2.2 (NMB1037), and glutathione synthetase, EC 6.3.2.3 (NMB1559). In turn, GSH can be converted to cysteine via gamma-glutamyltranspeptidase, EC 2.3.2.2 (NMB1057), and aminopeptidase N, EC 3.4.11.2 (NMB1416), yielding a functional γ-glutamyl cycle (Figure 3). This cycle helps to maintain the redox balance [64]. GSH can be oxidized to glutathione disulfide (GSSG) by glutathione peroxidase, EC 1.11.1.9 (NMB1621), thereby controlling the cellular hydrogen peroxide level [65].

In solution, cysteine can be converted chemically to cystine. Yu and DeVoe [66,67] corrected for this so-called auto-oxidation and suggested that electrons from cysteine enter the electron-transport chain at the flavoprotein level in a manner similar to those from succinate and NADH. They even suggested the presence of a specific cysteine oxidase, but no gene encoding this enzyme has been annotated in the MenB genome and no additional evidence supporting this hypothesis was found in the literature. It seems plausible that cysteine can increase the protonmotive force to drive oxidative phosphorylation. During oxidation of cysteine to cystine, electrons from cysteine are transferred to oxygen, yielding reactive oxygen, O_2 _^-^, as shown in Figure 3. This reactive oxygen can reduce cytochrome *c*, which in turn can provide a source of electrons for cytochrome *cbb*_3_, which reduces oxygen to water, causing the concomitant generation of a protonmotive force, Δμ^H+^, and ATP [68]. In addition, cysteine might be used directly as an electron donor by cytochrome *cbb*_3 _but no evidence supporting this hypothesis was found in the literature.

#### Oxidative stress

The reactive oxygen can also be processed by superoxide dismutases - SodC present in the periplasm or SodB present in the cytosol, EC 1.15.1.11 (NMB0884, NMB1398, respectively), followed by catalase, EC 6.3.5.5 (NMB1849, NMB1855), to regenerate oxygen, or by glutathione peroxidase as described above (see Figure 3). This protection mechanism against oxidative stress has been studied extensively [65]. Seib and co-workers concluded that *N. meningitidis *SodB plays a key role in protection against oxidative killing. The *sodC *mutant of *N. meningitidis *used in their study was no more sensitive to oxidative killing than the wild type. Paradoxically, Wilks and co-workers [69] found that a *sodC *mutant is significantly less virulent, indicating that SodC contributes to the virulence of *N. meningitidis*, most probably by reducing the effectiveness of toxic oxygen host defenses. This was confirmed by Dunn and co-workers [70], who showed that SodC contributes to the protection of serogroup B *N. meningitidis *from phagocytosis by human monocytes/macrophages, with *sodC *mutant organisms being endocytosed in significantly higher numbers than wild-type organisms. *In vitro*, oxidative stress might be induced by a high dissolved oxygen concentration, possibly resulting in oxidative killing. Therefore, the dissolved oxygen concentration in the chemostat experiments was controlled at a low level of 30%.

#### Metabolism of macromolecules

The metabolism of pyrimidine bases and nucleosides in *N. meningitidis *has been reviewed and studied extensively [71]. Although some of the early work contradicts the current information from the MenB genome in terms of pathway description, the results indicate that the required routes for pyrimidine biosynthesis are available in the genome. Furthermore, the MenB genome contains all the genes encoding the enzymes for the biosynthesis of UMP. Activities of the enzymes for this biosynthetic pathway were found previously in cell-free extracts [72].

In Gram-negative bacteria, such as *N. meningitidis *and *E. coli*, the cell envelope consists of an outer membrane, a dense peptidoglycan layer, and a cytoplasmic or inner membrane. The outer membrane has an asymmetrical organization in which the outside layer is primarily composed of lipopolysaccharide (LPS) and proteins and the inside layer contains phospholipids [73]. The inner membrane has a symmetrical phospholipid bilayer stucture, holding proteins primarily responsible for regulating the flow of nutrients and metabolic products in and out of the bacterium [74].

Membrane phospholipids form a major constituent of the cell envelope of *N. meningitidis *and maintain the integrity of the outer membrane. Rahman and co-workers [75] summarized early studies conducted in the 1960s and 1970s that indicated that the major phospholipid component of membranes isolated from *N. gonorrhoeae *consisted largely of phosphatidylethanolamine (PE), with varying amounts of phosphatidylglycerol (PG), cardiolipin (CL), and lysophosphatidylethanolamine (LPE). Subsequently, the total cellular fatty acids and extractable cellular lipids of *N. meningitidis *isolates were found to be similar to those of gonococci. The precise structures of *N. meningitidis *phospholipids, including their fatty-acylation patterns, have been elucidated quite recently [75]. Interestingly, the latter study shows that a major fraction (about 11%) of the total phospholipids appears to be phosphatidate (PA).

Lipid biosynthesis can be divided into two parts: biosynthesis of the fatty acids that are responsible for the characteristic hydrophobicity of lipids and attachment of the completed fatty acids to *sn*-glycerol-3-phosphate (GL3P), followed by the addition and modification of the polar head groups to yield phospholipids [76].

The fatty-acid biosynthetic pathway in *N. meningitidis *is similar to that in *E. coli*. All genes, except for a homolog of the *E. coli *β-hydroxyacyl-acyl carrier protein (ACP) dehydrase FabA, are present in the MenB genome. The absence of a *fabA *homolog in the MenB genome is not unique, as summarized by Rahman and co-workers [75], who state that the production of unsaturated fatty acids in *N. meningitidis *and other species may proceed via different biochemical pathways to that of *E. coli*. Homologs to glycerol-3-phosphate acyltransferase (PlsB) and cardiolipin synthase (YbhO) from *E. coli *were not found in the MenB genome. The reported phospholipid compositions in *Neisseria *species [75,77-79] in which no, or only trace quantities of, CL were found can be explained by the absence of a homolog for *ybhO*, but the absence of a homolog for *plsB *is striking. Apparently the gene(s) encoding the enzyme responsible for the formation of 1-acyl-sn-glycerol-3-phosphate has not been found in *N. meningitidis*. In fact, only 38 of the 125 prokaryotic genomes reported [22] annotated *plsB *or *plsB *homologs, all classified as gamma proteobacteria. Although it is possible that the reaction proceeds via a different biochemical pathway from that in *E. coli*, it is incorporated in the present metabolic model to complete the biosynthetic pathway to phospholipids.

The overall phospholipid composition used in our study was based on the information provided by Rahman and co-workers [75] - 11% PA, 71% PE, and 18% PG. In their study, no evidence supporting the presence of LPE was found. Bos and co-workers [80] reported a neisserial gene *pldA *(NMB0464), encoding a phospholipase, and characterized it as a neisserial autolysin that acts after bacteria have stopped dividing. This is consistent with LPE only being measurable when cells are harvested in the late exponential growth phase or stationary growth phase.

LPS, a second major constituent of the cell-envelope of *N. meningitidis*, is often referred to as endotoxin and plays an important role in virulence. It is held responsible for the severe pathological effects that occur during invasive meningococcal disease [81]. LPS consists of three parts: a lipid A part containing unique hydroxy fatty-acid chains, a core oligosaccharide containing 3-deoxy-D-manno-octulosonate (KDO) and heptoses, and a highly variable sugar backbone. In *E. coli*, lipid A is essential for cell viability [82,83], whereas an LPS-deficient meningococcal strain remains viable [84]. All genes involved in the biosynthesis of the lipid A of LPS are present in the MenB genome. Unlike the *LpxA *acyltranferase, present in the biosynthetic pathway of *E. coli*, the MenB *LpxA *acyltranferase (NMB0178) favors the substrate 3-OH C12 acyl-ACP [85], yielding a lipopolysaccharide structure as described earlier [86]. The HB-1 strain used in this study lacks expression of *galE *as a result of the deletion made into the capsule (*cps*) locus [87], leading to the synthesis of galactose-deficient LPS [88].

Heterogeneity in the LPS sugar backbone can be caused by phase variation of the genes involved [89], but phase-variable genes have not been found in the lipid A biosynthetic pathway. Kulshin and co-workers [90] found minor fractions of penta- and tetra-acylated lipid A structures in *N. meningitidis*, but the hexa-acylated structure predominated. Heterogeneity in lipid A has been found before in other species. Rebeil and co-workers [91] found that a shift in growth temperature of the genus *Yersinia *induced changes in the number and type of acyl groups on lipid A, suggesting that the production of a less immunostimulatory form of LPS upon entry into the mammalian host is a conserved pathogenesis mechanism and that species-specific lipid A forms may be important for life cycle and pathogenicity differences. Other bacterial species, such as *Salmonella typhimurium *and *Pseudomonas aeruginosa*, are able to covalently modify their lipid A through the enzymes PagL and PagP [92,93], but homologs to PagP and PagL have not been found in meningococcal genomes [94]. Structural analysis by mass spectroscopy of lipid A from strain HB-1 (data not shown) revealed that a monophosphorylated form of the above described hexa-acylated lipid A was present.

The capsular polysaccaride (whose synthesis is directed by the *cps *locus) is an important virulence factor in meningococcal pathogenesis and contributes to the survival of *N. meningitidis *in the blood stream [95,96]. The biosynthesis of capsular polysaccharide in *N. meningitidis *was first described by Blacklow and Warren [97]. They found that, unlike in mammalian cells, in *Neisseria N*-acetylneuraminic acid (Neu5Ac) is synthesized from *N*-acetylmannosamine (ManNAc) and phospoenolpyruvate without phosphorylated intermediates. Neu5Ac is the most common form of sialic acid in humans and plays an important role in intercellular and/or intermolecular recognition [98], explaining the difficulty of developing a safe and effective polysaccharide-based vaccine. Gotschlich and co-workers [99] determined the composition and structure of meningococcal group B capsular polysaccharide and found that it is composed of α-2,8-polysialic acid polymer chains, which are integrated in the outer membrane by a phospholipid anchor that is attached to the reducing end of the carbohydrate chain. This phospholipid anchor may help stabilize the outer membrane of the meningococcal mutant without endotoxin [84]. As mentioned above, the strain used in our study lacks the *cps *locus, as confirmed by Bos and Tommassen [100]. We therefore removed genes involved in sialic acid biosynthesis and polysaccharide transport from our model, as well as the *galE *gene. Subsequently, the *rfbB*, *rfbA *and *rfbC *genes located downstream of *galE*, which code for enzymes involved in the biosynthesis of dTDP-rhamnose, were also removed, yielding a dysfunctional biosynthetic pathway. Hence, these pathways were not included in the simplified model.

Peptidoglycan forms the third major constituent of the cell envelope of *N. meningitidis*. Antignac and co-workers [101] determined the biochemical structure of peptidoglycan in various *N. meningitidis *strains in detail and found that it consists of a maximum of two layers. Variations in the degree of cross-linking and O-acetylation appeared to be associated with the genetic background of the strains. The percentage of crosslinking of the peptidoglycan was around 40%, which is consistent with that determined for other Gram-negative bacteria [102-105], whereas the percentage of O-acetylation per disaccharide was on average 36%. O-acetylation of peptidoglycan results in resistance to lysozyme and to other muramidases [106], suggesting that nonspecific lysis of the bacteria in the host enviroment by lysozyme can be prevented. Other studies show that peptidoglycan structures are recognized by the innate immune system [107,108]. Consequently, O-acetylation might contribute to affect the proper response to infection. Most of the strains analysed by Antignac and co-workers [101] predominantly contained muropeptides carrying a tetrapeptide chain, but di-, tri- and pentapeptide chains were also found. Their analysis also showed that none of the muropeptides carried glycine residues on the peptide backbone, as has been observed in gonococci [109,110]. Hence, meningococci only synthesize D-alanyl-meso-deaminopimelate cross-bridges. However, the gene involved in alanyl-meso-deaminopimelate cross-bridging for biosynthesis of the peptidoglycan polymer structure has not been annotated. The enzyme involved in glycine cross-bridging of peptidoglycan (EC 2.3.2.10) is not present in the MenB genome, thus confirming the observations of Antignac and co-workers [101]. The biosynthetic pathway for peptidoglycan biosynthesis in our metabolic model includes the information provided by Antignac and co-workers [101], using 36% O-acetylation per disacharride and 40% crosslinking, whereas the model muropeptide only contains the predominant tetrapeptide backbone.

### Main characteristics of the genome-scale model

The main characteristics of the genome-scale metabolic network are summarized in Table 1. The MenB genome contains 2,226 ORFs of which 2,155 are protein encoding genes, 59 are tRNA encoding genes and 12 are rRNA encoding genes [111]. At present, 1,307 genes from the total of 2,155 protein-coding genes have an annotated function (60.6%), of which 146 genes encode transporter functions [112].

**Table 1:** Main characteristics of the genome-scale metabolic network of *N. meningitidis*

ORFs	555
Annotated functions	509
Annotated putative functions	46
Unannotated functions	38
	
Metabolites	
Unique intracellular metabolites	471
Extracellular metabolites (minimum, based on measurements)	33
	
Reactions	496
Intracellular reactions	451
Transport fluxes	74
Biosynthesis of macromolecules and biomass assembly	5

For construction of the genome-scale model, a total of 555 ORFs were considered, corresponding to at least 496 associated reactions (including membrane-transport reactions) and at least 471 unique metabolites. The exact number of metabolites cannot be determined accurately because of the presence of polymerization reactions in which numerous intermediate compounds can be synthesized. In cases where numerous ORFs accounted for a single reaction for example, the various subunits of ATPase) the reaction was counted once, which explains the lower reaction count compared to other genome-scale networks [14]. To complete the metabolic network, two chemical oxidation reactions were added based on the literature, four reactions were added to account for biosynthesis of the macromolecules DNA, RNA, protein and lipid, one reaction was added for biomass assembly and 38 reactions were added to fill pathway gaps (unannotated functions in Table 1). For these unannotated functions, a corresponding gene has not been found in *N. meningitidis*. Furthermore, for nine of these functions a corresponding gene has never been found in any organism. The complete reaction database along with the genes involved, enzyme numbers and metabolites can be consulted in Additional data file 1. A detailed description of the biomass composition and its biosynthesis is in Additional data file 2.

### Construction of a simplified metabolic model

The genome-scale model was simplified to the model shown in Figure 2. Simplification was carried out purely for ease of understanding and was done as follows. First, successive reactions in a linear pathway were lumped up to the first branch point. Second, some reactions were neglected (for example, biosynthesis of amines, cofactors and vitamins) because the production rate of these metabolites is very small in comparison with the production rate of macromolecules required for biomass assembly. Third, reactions were omitted to prevent 'dead ends'. A dead end exists in a metabolic network if a metabolite is at the end of a metabolic pathway and, based on the literature and our own measurements, the metabolite does not accumulate in biomass nor is it excreted to or taken up from the medium. Examples in our case are hydroxypyruvate and lactaldehyde. In reactions that can use NADH or NADPH as co-factors, the NADH co-factor was used in the model unless stated otherwise in Additional data file 1. In the approach used, a distinction between NADH and NADPH preference cannot be made. Additional enzymatic analysis to distinguish between NADH or NADPH preference is complicated because of the presence of transhydrogenase. The final simplified model used for flux-balance analysis included 161 reactions (129 intracellular reactions, 33 transport fluxes) and 131 intracellular metabolites and can be consulted in Additional data file 1.

### Modeling of the metabolic network

In metabolism, substrates are converted into the different macromolecules that together make up biomass. Thus, the macromolecular composition of biomass determines the flux distribution, and a shift in the macromolecular composition of biomass will result in a shift in the flux distribution. Consequently, experimental determination of the biomass composition is very important in mathematical modeling of cellular metabolism, as described in detail elsewhere [113,114]. The measured concentrations of substrates, biomass and products, which can be found in Additional data file 2, were converted to measured conversion rates using mass balances.

#### Monte Carlo simulation

**Figure 4:**
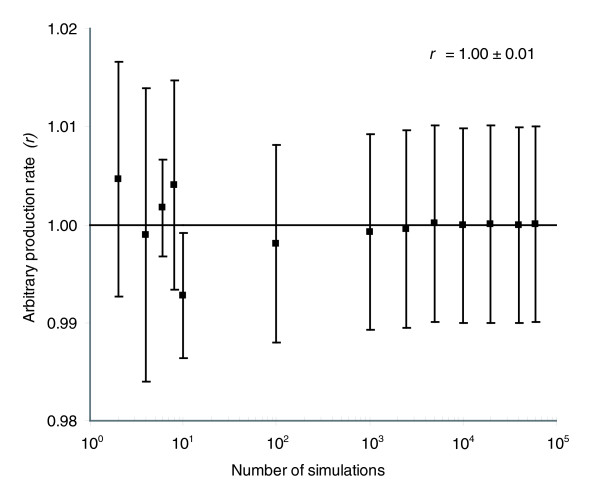
Determination of measurement variance using Monte Carlo simulation. When an arbitraty value (*r*) for the production rate of a hypothetical product of 1.00 with standard deviation of 0.01 was used as input for Monte Carlo simulation, 10^4 ^simulations were required to obtain the original input value (1.00 ± 0.01), showing that accurate results for the actual measured input values can be expected after 10^4 ^simulations.

Before error diagnosis was performed, errors in the primary measurements were translated to errors in measured conversion rates using a Monte Carlo approach [115,116]. In complex mass balance equations (for example, CO_2 _production rate) in which various measured values with various standard deviations are included, determination of the total variance is quite laborious using standard error propagation. Therefore, variances of the measured conversion rates of the various substrates and products were calculated using Monte Carlo simulation. The exact procedure involved mass balances that were formulated for the reactor configuration and is described in detail in Materials and methods. Accurate results were obtained after 10,000 simulations, as shown in Figure 4. The resulting average measured conversion rates and their corresponding variances were then used as input for flux-balance analysis. In addition (data not shown), all separate simulated measured conversion rates were also used directly as input for flux-balance analysis, resulting in 10,000 flux distributions. The final average flux distribution, calculated from these 10,000 distributions, was identical to the one obtained using the average measured conversion rates as input. All calculated conversion rates as well as the fluxes appeared to be normally distibuted.

#### Error diagnosis and balancing

Laws of conservation result in a number of linear constraints on the measured conversion rates of the various compounds. The measured conversion rates and their corresponding variances, which were calculated using Monte Carlo simulation, were subjected to gross error diagnosis. First the redundancy matrix, *R*, was calculated as described previously [117]. Matrix *R*, expressing the conservation relations between the measured conversion rates only, contained two independent equations. Inspection of these equations indicated a carbon and a nitrogen balance. The residuals obtained after multiplication of the redundancy matrix with the measured conversion rates could be explained on the basis of random measurement variances [118] with test values of *h*_*e *_= 1.598 and *h*_*e *_= 1.391 for the first and second experimental set of measured rates, respectively, where the 95% chi-square critical value is 5.992. Also, the individual carbon and nitrogen balance could be closed for both datasets. Thus, the measurements contained no gross errors and the model is also valid with respect to the measurements. The results of the statistical test are shown in Additional data file 1. Hence, the measured rates were balanced by minimizing the square of the distance between the actual measurement and the adjusted measurement with the measurement variance as a weighing factor as described previously [117]. The resulting balanced measurement rate vector, which is more accurate than the primary measurement rate vector, was used to calculate the unknown fluxes and conversion rates (see Additional data file 1). The combinations of fluxes and unknown exchange rates that cannot be uniquely identified (that is, calculated from the measurements) were isolated using singular value decomposition. These underdetermined parts of the simplified network are listed in Additional data file 1.

#### Flux distribution

As stated, the network contains underdetermined parts, for which the flux values cannot be calculated from the measured rates. To calculate a solution for these fluxes, constraints can be set on certain enzymatic reactions on the basis of available biochemical and thermodynamic literature. In addition, objective functions and linear optimization can be used to calculate fluxes and unknown rates in the underdetermined part(s) of the network, as explained in Materials and methods. The main objection to the technique of linear programming to find a solution for an underdetermined part of the network is that the objective function may not be valid for the biological system. The minimum norm constraint is an optimization function that minimizes the length of the solution vector without any further restrictions, as explained in more detail in Materials and methods, and was used to calculate fluxes and unknown rates in the underdetermined part(s) of the network. Like Bonarius [119], we hypothesize that the minimum-norm constraint correctly assumes that the total flux activity is minimized in order to fulfil the efforts of the bacteria to attain an efficient flux distribution.

The flux values are given in Additional data file 1. For most of the underdetermined fluxes realistic values are obtained using the minimum norm solution. For instance, the calculated flux ratio between the ED cleavage and the PP pathway shows that the major part of pyruvate (69 ± 6%) is synthesized through the ED cleavage, which is in agreement with the literature [32]. Furthermore, we calculated that 91 ± 7% of the NADPH that is produced in both the PP-pathway (flux 17 and 18) and the TCA cycle (flux 2) is used for biosynthesis while 9 ± 7 % is converted to NADH using transhydrogenase (flux 74). Some of the flux values obtained for underdetermined fluxes using the minimum-norm constraint are unlikely, and are clearly an artifact of the minimum-norm solution. These are the positive sulfate production rate and the negative flux from pyrophosphate to phosphate (flux 71). By measuring thiosulfate, sulfate, and H_2_S, these underdetermined fluxes become determined and this problem can be solved. It must be emphasized that the actual flux distribution through the underdetermined parts may very well be different from the minimum-norm solution. Only isotopic tracer experiments can deliver true flux values for some underdetermined parts, like, for example, the ED and PP pathway. Nevertheless, the calculated flux distribution in the present study also shows that it is in agreement with the literature for specific underdetermined parts of the network.

### Application of the model for process development purposes

The metabolic model presented in this paper offers a framework to study *N. meningitidis *metabolism as a whole or certain aspects of it. For example, gene deletion analysis could be carried out to study which genes are essential for growth in the host environment, which in turn could serve to identify possible targets for new antibiotics. In the present study, apart from the data analysis discussed above, the model was developed for process development purposes. In particular, the model was used for the design of minimal medium for growth of *N. meningitidis*.

#### Design of minimal medium

The amount of available literature describing the requirements for growth of *N. meningitidis *is tremendous, which provided an easy starting point for designing a minimal growth medium. The MenB genome was next checked for the presence of the membrane-transport functions that are required for utilization of substrates. In the case of carbon, nitrogen, and sulfur substrates the genome was also checked for the presence of subsequent processing pathways.

The membrane-transport functions for utilization of the inorganic ions Na^+^, K^+^, Mg^2+^, Cl^-^, and PO_4 _^3 ^are annotated in the MenB genome. Genes for membrane transport of the trace elements Fe^3+^, Cu^2+^, Zn^2+^, Co^2+^, Ca^2+^, and Mn^2+ ^are also annotated. These ions often function as cofactors for metal-activated enzymes or metalloenzymes. For instance, copper-zinc superoxide dismutase is a metalloenzyme that uses copper and zinc to help catalyze the conversion of superoxide anion to molecular oxygen and hydrogen peroxide. Furthermore, aconitase (a TCA enzyme) contains several iron atoms bound in the form of iron-sulfur clusters, which participate directly in the isomerization of citrate to isocitrate. The only trace element added as a supplement to the medium that could not be related to an annotated function in the genome was molybdenum. In general, molybdenum is necessary for the activity of several enzymes [120] and is added as trace element in media for growth of various bacteria, such as *E. coli *[121]. For this reason MoO_4 _^2- ^was added to complement the minimal medium. However, it remains unclear whether molybdenum is an essential requirement for growth of *N. meningitidis*.

*N. meningitidis *is able to metabolize a variety of sugars and amino acids for biosynthesis of macromolecules and their precursors as indicated by the presence of the relevant transporter genes and the available processing pathways (ED pathway, PP pathway, amino-acid biosynthesis). The main utilizable carbon sources include glucose, sucrose, lactose, fructose, maltose, gluconate, lactate, and pyruvate. An amino acid like glutamate can serve as both a carbon and a nitrogen source, as the glutamate deamination product (α-ketoglutarate) can be metabolized via the citric acid cycle. However, in the literature no media were found in which glutamate was used as the sole carbon and nitrogen source for growth of *N. meningitidis*. A possible disadvantage of using other amino acids as carbon and/or nitrogen sources is that the deamination might result in acid products that cannot be processed metabolically and will therefore accumulate. To select the best carbon source, the metabolic model was used to simulate growth on different carbon sources *in silico*. In all *in silico *experiments, ammonium was selected as the sole nitrogen source based on the presence of the ammonium transporter Amtb (NMB0615) and the NADP-specific glutamate dehydrogenase processing pathway (NMB1710).

For the *in silico *simulations, the uptake of the carbon source was fixed at a certain value and biomass formation was optimized. Furthermore a maintenance requirement of 2.81 × 10^-3 ^mol/gram/hour was set, which was obtained from the literature [122]. The predicted yields of biomass on various carbon substrates are given in Table 2. The metabolic model predicted that glucose is the preferred carbon substrate for growth of *N. meningitidis *in terms of biomass yield. Accordingly, glucose was selected as sole carbon source for growth. When growth was simulated using glucose as the carbon source, the predicted C:N consumption ratio was 9:1, indicating the minimal amount of ammonium that is required in the growth medium. A wide range of sulfur-acquisition routes is available in *N. meningitidis*. Both thiosulfate [63] and sulfate [54] were selected as sulfur sources in the growth medium. *In silico *predictions showed that thiosulfate is the preferred sulfur source for growth, which can be explained by the fact that less energy is required for utilization of thiosulfate in comparison with sulfate. The results of these *in silico *experiments are listed in Additional data file 1.

**Table 2:** *In silico *yield, Y_xs _of biomass on substrate for growth on different carbon sources

Substrate	*Y*_xs _*in silico *(grams per mole carbon (Cmol))
Glucose (GLC)	10.7
Lactate (LAC)	9.6
Glutamate (GLU)	8.2
Acetate (ACE)	0.0 (not possible)
GLC + LAC (50/50 Cmol/Cmol)	10.4
GLC + GLU (50/50 Cmol/Cmol)	9.7
LAC + GLU (50/50 Cmol/Cmol)	9.0
ACE + GLU (50/50 Cmol/Cmol)	6.3

**Figure 5:**
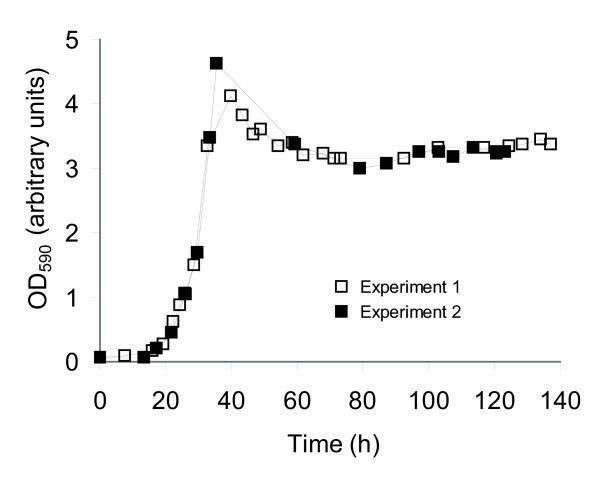
Growth of *N. meningitidis *in a minimal medium designed from the model. Growth of *N. meningitidis *strain HB-1 in minimal medium in a bioreactor operated in chemostat mode after an initial batch phase of approximately 36 h were assessed by optical density. Two experiments in identical medium and growth conditions are shown here. Conditions were as described in Materials and methods.

Constraint-based models give information on which substrates to use and, for some substrates, in which ratio they should be used. However, they give no information on the actual concentrations that should be used. For our study the required concentrations (by order of magnitude) of the various compounds in the minimal medium were derived from the relevant literature referred to earlier. The resulting minimal medium (see Table 3) is suitable for growth of *N. meningitidis *(Figure 5). To obtain higher biomass concentrations at the end of a batch culture further optimization of the concentrations is needed.

**Table 3:** Medium composition

Component	Concentration
NH_4_Cl	23.5
NaCl	102
MgSO_4_.7H_2_O	2.43
K_2_HPO_4_	12.5
KH_2_PO_4_	4.58
FeCl_3_.6H_2_O	0.30
Trace-element solution (ml/l)	2.0
D-Glucose monohydrate	31.1
Na_2_S_2_O_3_.5H_2_O	0.38

All values in mM unless stated otherwise.

## Conclusion

*In silico *genome-scale flux models have been proved to give insight into the complexity of metabolism and can be used succesfully as tools for medium design and metabolic engineering strategies. Many studies lack sufficient experimental datasets to check the consistency of the measurements and the validity of the metabolic network model. In the present study, the experimental datasets used in flux-balance analysis proved sufficient to check for the presence of gross measurement errors or errors in the model, neither of which was found to be present.

Working from the genomic database of MenB together with biochemical and physiological information provided in the literature, a genome-scale flux model for vaccine process development purposes was constructed. From these information sources a minimal medium for growth was designed and tested succesfully in batch and chemostat cultures. The simplified metabolic network that was derived from the genome-scale metabolic network was verified using flux-balance analysis in two duplo chemostat cultures. The specific rates, being the input of the model, were generated from the measured concentrations using Monte Carlo simulation. The model contains underdetermined parts, for which fluxes were calculated using the minimum-norm constraint, providing a possible solution for these underdetermined parts. Despite the unlikely positive sulfate production rate and the negative flux from pyrophosphate to phosphate, which are both underdetermined parts of the network, the flux distribution for the remaining underdetermined parts seems plausible. This is strengtened by the observation that the major part of pyruvate (69 ± 6%) is synthesized through the ED cleavage, which is in good agreement with the literature [32].

Methods for integration of metabolic network information with gene-expression profiles (transcriptomics) were described recently [123-125]. Such studies bridge the gap between the putative, homology-based information provided by genomes and the actual situation, and greatly contribute to further improving our understanding of cellular processes and function.

## Materials and methods

### Strain

*N. meningitidis *strain HB-1, a non-encapsulated, non-piliated variant of the group B isolate H44/76 [100], was used throughout this study. Stock cultures of strain HB-1 were stored at -135°C and when required, a 500-ml shake flask, containing 150 ml of the chemically defined medium described below, was inoculated. After approximately 8 h of incubation at 35°C with shaking at 200 rpm in an aerobic humid atmosphere, the culture was used to inoculate the medium in the bioreactor.

### Medium

The composition of the chemically defined medium described in this study is based on the information provided by the genome and the literature as descibed in the text. The composition of the minimal medium is given in Table 3. The trace-element solution contained (values in mM) CaCl_2_.2H_2_O, 68; ZnSO_4_.7H_2_O, 0.17; Na_2_MoO_4_.2H_2_O, 0.08; MnCl_2_.4H_2_O, 0.4; CoCl_2_.6H_2_O, 0.04 and CuSO_4_.5H_2_O, 0.04. The medium was sterilized by filtration (0.22 μm pore size). To prevent precipitation, the minimal medium and a sterile solution containing FeCl_3_.6H_2_O in 0.6 M HCl were added separately to the reactor using two pumps (Watson-Marlow Bredel, Falmouth, UK).

### Chemostat cultures

Bacteria were grown in a 3-l autoclavable ADI bioreactor (Applikon, Schiedam, The Netherlands), operated in chemostat mode, with a working volume of 1.4 l. Temperature, pH, dissolved oxygen (DO) concentration, and stirrer speed were contolled at 37°C, 7.0, 30%, and 600 rpm, respectively. The total gas flow rate was kept constant at 1.0 l/min. The oxygen concentration was controlled by changing the oxygen fraction in the gas flow using headspace aeration only. The growth rate was controlled at 0.04/h. After at least three residence times, physiological steady state was assumed based on online measurements (constant DO signal, O_2 _and CO_2 _concentration in the off-gas) and offline measurements (constant optical density, zero glucose concentration). In steady state, a 1.0-l sample was taken and divided into portions. After centrifugation of the portions (3000 *g*, 1 h at 4°C) cells were washed once with saline solution (0.85 w/v % NaCl) and centrifuged again. After centrifugation, the saline solution was discarded and the pellet was freeze-dried without additives (Leybold, Cologne, Germany). The freeze-dried pellet was stored at -80°C until further analysis. The chemostat cultures were perfomed in duplicate.

### Analytical procedures

#### Biomass concentration

Dry biomass concentration was determined in fourfold for each sample by centrifugation (8000 *g*) of 50.0 ml of culture broth in preweighed tubes. The cell material was dried at 80°C for at least 24 h. Before weighing, the tubes were cooled in a desiccator for at least 1 h. Dry cell weight was corrected for salts present in the medium.

#### Off-gas analysis

The oxygen and carbon dioxide concentrations in the exhaust gas from the chemostat cultures were measured with a mass spectrometer (Prima White Box 600, Thermo Electron, Winsford, UK).

#### *k*_*L*_*a *determination

The volumetric oxygen transfer coefficient, *k*_*L*_*a*, in the bioreactor was determined accurately at 37°C using a steady-state setup and a phosphate-buffered saline solution (NVI, Z3000) as reference liquid. The steady-state determination method makes use of the global balance of oxygen over the bioreactor in the gas phase and liquid phase. The calculated values were confirmed using the dynamic method with correction of electrode response time [126].

#### Metabolite concentrations

Glucose and lactate were determined with a YSI 2700 glucose/lactate analyser (Yellow Springs Instruments, Yellow Springs, OH). Ammonium was determined with an enzymatic kit (Boehringer, Mannheim, Germany). Acetate, ethanol and other possible metabolites present in the culture supernatant were determined by ^1^H-NMR using a Jeol JNM ECP 400 spectrometer operating at 400 MHz (JEOL, Tokyo, Japan) equipped with a JEOL stacman autosampler for 16 samples. Culture supernatant was analyzed by adding 0.1 ml of D_2_O containing 3-(trimethylsilyl) [D_4_]propionic acid sodium salt (TMSP, 0.167 mM) to 0.9 ml sample. The water signal was suppressed by irradiating the signal with standard NMR software. The spectra were referenced using the TMSP signal at 0 ppm. Metabolite concentrations were quantified by integration of the relevant signals.

#### Protein

The amino-acid composition of biomass protein was determined after hydrolysis (6 M HCl, 24 h, 110°C) and subsequent amino-acid analysis using an HPLC method as described previously [127]. Free amino acids in the culture supernatant were determined using the same method. Cysteine and tryptophan, which were destroyed during acid hydrolysis, were calculated on the basis of the predicted amino-acid composition in the genome using ratios as described in Additional data file 2. Glutamine and asparagine were converted to glutamate and aspartate, respectively, during acid hydrolysis and were also calculated on the basis of the predicted amino-acid composition in the genome (see Additional data file 2). Total biomass protein was calculated by summation of the measured amino-acid concentrations and corrected for salt present in the freeze-dried biomass. No correction was done for amino acids present in peptidoglycan. The methods recovery was determined as 0.85 ± 4%, based on measurements of pure bovine serum albumin and corrections were made accordingly. The measured amino-acid composition and the assembly into protein can be found in Additional data file 2.

#### Fatty acids

The amount of total fatty acids and the fatty-acid composition were analyzed using a modified gas chromatography method [128]. Saponification reagent (1.2 ml of 3.75 M NaOH in 50/50 v/v methanol/water) was added to 12 mg freeze-dried biomass to liberate the fatty acid from cellular lipid. After vortex mixing for 10 sec the samples were placed at 100°C for 30 min. After cooling for 15 min to room temperature, 2.4 ml of methylation reagent (3.25 M HCl in methanol) was added and after vortex mixing for 10 sec the samples were placed at 80°C for 20 min. After cooling down to room temperature, the fatty-acid methyl esters were extracted by adding 1.5 ml hexane reagent (50/50 v/v hexane/methyl tertiary butyl ether). Samples were mixed for 15 min and centrifuged (3 min, 1,834 *g*). The aqueous layer was removed and the organic phase was washed by adding 3.0 ml washing agent (0.3 M NaOH). The samples were mixed for 5 min and centrifuged again (3 min, 1,834 *g*). The organic phase was finally transferred to a gas chromatograph vial and analyzed using a 6890 Agilent gas chromatograph [129]. The standards and samples were injected into the gas chromatograph equipped with a flame ionization detector via an automated sequence run. An Agilent 19091B-102 capillary column (Ultra 2; 5% Phe-methylsiloxane; 25 m × 0.200 mm; film thickness 0.33 μm) was used. Oven temperature was programmed as follows: 60°C, hold 3 min; 280°C at 25°C/min, hold 4 min; 320°C at 15°C/min, hold 2 min; 340°C at 20°C/min, hold 0 min). The injector temperature was set at 275°C and detector temperature was set at 375°C. Helium was used as carrier gas at a constant flow-application of 2.0 ml/min. Fatty-acid methyl esters were identified by their retention times in comparison to those of a commercial standard (Microbial ID, Inc., Newark, DE, USA). Calibration curves of pure fatty acids present in *N. meningitidis *were made based on the identified fatty acids [128] and confirmed in literature [130]. The pure fatty acids used as calibration standards were dissolved in dichloromethane (Sigma, St Louis, MO). All fatty acids were purchased from Sigma, except for palmitelaidic acid (C16:1-trans 9) which was purchased from ICN Biomedicals (MP Biomedicals, Irvine, CA). Hydroxy-fatty acids were quantified using C12:0-2OH as an internal standard, whereas non-hydroxy-fatty acids have been quantified using C15:0 as an internal standard. The measured fatty-acid concentrations were corrected for salt present in the freeze-dried biomass. The measured fatty-acid composition and the assembly into lipid can be found in Additional data file 2.

#### Lipopolysaccharide

LPS was isolated by the hot phenol-water extraction as described before [131]. Isolation and structural analysis of lipid A was performed by nanoelectrospray tandem mass spectrometry on a Finnigan LCQ in the positive ion mode as described previously [132]. LPS was quantified on the basis of the measured amount of C12:0-3OH (see Fatty acids).

#### RNA and DNA

The biomass RNA content was determined as described previously [133] and the biomass DNA content was determined colorimetrically as described previously [134]. The DNA composition was derived from the complete nucleotide composition in the genome sequence. For RNA the uridine content was based on the thymine content in the genome sequence [23]. The measured concentrations were corrected for salt present in the freeze-dried biomass.The calculated RNA and DNA composition and their assembly can be found in Additional data file 2.

#### Biomass composition

Biomass biosynthesis was set as a linear combination of the macromolecules protein, DNA, RNA, lipid, peptidoglycan and LPS, which were considerd to account for the overall biomass composition. The energy requirement for biomass assembly was also considered and estimated to be 13.27 mol ATP/mol biomass. This value was calculated using linear regression of available 1/*Y*_x/ATP _versus growth-rate data from *E. coli *[122]. The accuracy of the estimation is, however, quite low. A detailed calculation of the biomass composition and its assembly, together with an overview of the metabolites measured in the culture supernatant and off-gas can be found in Additional data file 2.

### Modeling

#### Mathematical formulation

The genome-scale metabolic network was first simplified as described in the text. Next, the stoichiometric matrix was constructed from the set of reactions using a self-made computer program running in Visual Basic (Microsoft, Seattle, WA). Both Monte Carlo simulation and flux-balance analysis were performed in self-made computer programs running in Matlab (version 6.5 r13; Mathworks, Natick, MA).

Methods for solving a metabolic network or set of linear equations are discussed extensively elsewhere [135-138]. A brief summary is given below. In steady-state growth, the reactions of the considered metabolites form a set of linear equations that can be expressed in matrix notation as:

$$\text{A}\cdot \nu =\text{A}\cdot \left(\begin{array}{c} \text{x}\\ \text{r} \end{array}\right)=0$$

where A (*m *× *n*) is the stoichiometric matrix which contains *m *metabolites and *n *reactions, including (measured) exchange reactions, and ν is the flux vector that contains the unknown fluxes *x *and (measured) exchange rates *r*. A solution to the metabolic network will exist provided that A is nonsingular. Singularities in A may arise due to reaction dependence or network observability problems. Equation (1) states a compound balance for each of the metabolites in the system. If measurements are made of substrate and product concentrations, and the conversion rates of these metabolites are calculated, such a set of measured rates should match Equation (1).

#### Error diagnosis and balancing

Methods for error diagnosis and balancing are discussed extensively elsewhere [117,118]. A brief summary is given here. To be able to do error diagnosis, the measurement set should contain balanceable rates. Balanceable rates can be found by calculating the redundancy matrix [117], which contains linear relations between measured rates that are thus balanceable. Parts of the relations are usually formed by a carbon, nitrogen, and redox balance. In practice, all measurements suffer from measurement noise, and sometimes process noise, which leads to unavoidable inaccuracies in the measured conversion rates. Consequently, the product of the redundancy matrix and the measured rates is not zero, but yields a vector of residuals, ε:

R·r_m _= ε

where *R *is the redundancy matrix and and *r*_m _the measured conversion rate vector.

To test whether ε can be explained from random measurement errors, the following test function was developed [139]:

$${\text{h}}_{\text{e}}=\varepsilon^{\text{T}}\cdot \psi_{\varepsilon }^{-1}\cdot \varepsilon$$

where *h*_e _is the test function and ψ_ε _is the variance-covariance matrix of the residual vector ε, which is calculated from the variance-covariance matrix ψ_δ _of the measurements according to:

ψ_ε _= R·ψ_δ_·R^T^

when ε is due to random measurements only, *h*_e _follows a χ^2^-distribution with a number of degrees of freedom equal to the rank of ψ_ε _[118]. When no gross errors are detected the measured rates should be balanced to obtain more reliable intracellular fluxes

r_t _= r_m _- δ

where r_t _is the vector containing the balanced specific production rates (mol/g/h) and δ is the vector containing the deviations between measured and balanced rates, which can be calculated using a weighed minimum last square approach according to the following equation:

$$\delta =\psi_{\delta }\cdot {\text{R}}^{\text{T}}\cdot \psi_{\varepsilon }^{-1}\cdot \varepsilon$$

The resulting balanced measurement rate vector can be used to calculate the unknown fluxes and production rates. As described in more detail below, Monte Carlo simulation can be used to determine the variance of a measured conversion rate.

#### Network sensitivity analysis

The matrix containing the mass balances over the intracellular compounds can be checked for network sensitivity problems by performing singular value decomposition (SVD). SVD is a mathematical technique that decomposes matrix A into three matrices as follows:

A = U·W·V^T^

The matrix W is an orthogonal matrix containing the eigenvalues of the matrix. The columns of U, whose corresponding elements of W are non-zero, form an orthonormal basis that spans the range of A. The columns of V, whose corresponding values of W are zero, are an othonormal basis for the nullspace of A. In other words, the columns of V contain the combination of fluxes and rates that cannot be uniquely identified. Here we use SVD to identify and isolate the combinations of fluxes and unknown exchange rates that cannot be uniquely identified (that is, calculated from the measurements) [138]. It is worth noting that no measurements are needed to perform this check. If underdetermined parts are present, the number of solutions for the flux vector is infinite.

To reduce the number of allowable solutions for the underdetermined parts of the metabolic network, limits on the range of individual flux values can be set on the basis of available biochemical and thermodynamic literature for the enzymatic reactions. These constraints have the form:

α ≤ ν ≤ β

where α and β are the lower and upper limits, respectively. Thermodynamic constraints regarding the reversibility or irreversibility of a reaction can be applied by setting α for the corresponding flux to zero. To further shrink the original solution space to a single solution, linear optimization can be used to find the solution that optimizes a particular objective function. Some examples of objective functions are those that maximize biomass formation or minimize the production of ATP, NADH, NADPH, or a particular metabolite [135,140].

An alternative objective function minimizes the sum of the squares of the fluxes, and is called the minimum-norm constraint. The minimum-norm constraint minimizes the length of the solution vector without any further restrictions using the Moore-Penrose-pseudo-inverse of A [141]. The Moore-Penrose-pseudo-inverse gives the one solution vector that has the smallest Euclidian norm (for example, the smallest square of its length). Like Bonarius [119], we hypothesize that the minimum-norm constraint correctly assumes that the total flux activity is minimized in order to fulfill the efforts of the bacteria to achieve an efficient flux distribution.

#### Monte Carlo simulation

Error diagnosis and balancing are important aspects of flux-balance analysis. Before error diagnosis can be done, errors in the primary measurements need to be translated to errors (that is, variances) in measured conversion rates. This can be done using a Monte Carlo approach [115,116] as follows: The various measured values and their standard deviations were used as input to calculate the measured conversion rate of a specific metabolite numerous times. The value of each measured variable in the mass balance equation of this specific metabolite was simulated randomly within the allowed standard deviation interval of each variable. For example, the mass balance describing the conversion rate of glucose contained four measured variables: the glucose concentration in the incoming and outgoing medium, the medium flow, and the biomass concentration. In each Monte Carlo run, a random value was assigned to each measured variable within the allowed standard deviation interval of that variable. This was done separately for all of the four measured variables. The resulting four random values were used to calculate the conversion rate of glucose. After numerous simulation runs, the average glucose conversion rate and the corresponding variance was calculated from all the simulated conversion rates. As stated in the text, accurate results were obtained after 10,000 simulations, resulting in an average measured conversion rate with a normal error distribution (a prerequisite for part of the flux-balance analysis procedure). All other measured conversion rates were calculated similarly, resulting in a set of measured conversion rates and corresponding variances that were subjected to error diagnosis.

#### In silico modeling

All *in silico *experiments were done using the simplified metabolic model. All simulations were performed in a self-made computer programs running in Matlab (version 6.5 r13). The flux through the non-growth-associated ATP-maintenance reaction was fixed to 2.8 mmol/gram dry weight/h [122]. The lower limit of irreversible reactions was set to zero and fluxes through all other intracellular reactions had no upper or lower limit. In addition, the production rate of all amino acids and extracellular protein was set to zero. The following external metabolites were allowed to freely enter and leave the system: ammonia, water, phosphate, thiosulfate, sulfate, carbon dioxide, oxygen, and protons. In addition, acetate, hydrogen sulfide and ethanol were only allowed to leave the system. Growth on different carbon sources was simulated by allowing the cabon source under study to enter the system. The consumption rate of all carbon sources was fixed to 4.23 mmol C/gram dry weight/h. In all simulations, the maximization of biomass formation was used as an objective function.

## Additional data files

The following additional data are available online with this paper. Additional data file 1 is an Excel file that includes 7 worksheets. The first worksheet named 'genome-scale model' includes the complete reaction database along with involved genes, enzyme numbers and metabolites. The second worksheet named 'simplified model' contains the simplified metabolic model. The third worksheet named 'abbreviations' contains a list of abbreviations of the metabolites. The fourth worksheet named 'redundancy matrix' contains the redundancy matrices of the experimental data sets. The fifth worksheet named 'measured rates, balanced rates' contains a table in which the experimental set of measured rates and balanced rates is given. In addition, the results of the statistical acceptance test are given in this table. The sixth worksheet named 'calculated fluxes' contains the calculated flux distributions of both experimental data sets using the mimimum norm constraint. The seventh worksheet named 'in silico' contains the flux distributions of the *in silico *experiments. Additional data file 2 is a Word document that includes a detailed description of the biomass composition together with an overview of the metabolites measured in the culture supernatant and off-gas and references [142-145].

## Supplementary Material

Additional data file 1 The first worksheet named 'genome-scale model' includes the complete reaction database along with involved genes, enzyme numbers and metabolites. The second worksheet named 'simplified model' contains the simplified metabolic model. The third worksheet named 'abbreviations' contains a list of abbreviations of the metabolites. The fourth worksheet named 'redundancy matrix' contains the redundancy matrices of the experimental data sets. The fifth worksheet named 'measured rates, balanced rates' contains a table in which the experimental set of measured rates and balanced rates is given. In addition, the results of the statistical acceptance test are given in this table. The sixth worksheet named 'calculated fluxes' contains the calculated flux distributions of both experimental data sets using the mimimum norm constraint. The seventh worksheet named 'in silico' contains the flux distributions of the *in silico *experiments. Click here for file

Additional data file 2 A detailed description of the biomass composition and and overview of the metabolites measure in the culture supernatant and off-gas. Click here for file
