# Supplementary material for: Modeling Neisseria meningitidis metabolism: from genome to metabolic fluxes
Source: Genome Biol. 2007 Jul 6;8(7):R136. doi: 10.1186/gb-2007-8-7-r136 (PMC2323225; doi:10.1186/gb-2007-8-7-r136)
Supplement: Additional data file 2 — A detailed description of the biomass composition and and overview of the metabolites measure in the culture supernatant and off-gas. [file gb-2007-8-7-r136-S2.doc]

# Biomass composition

#### S1A Overall cellular composition of N. meningitidis

In a specific medium for growth, the overall cellular composition of bacterial cells depends on the growth rate of the cells [142, 143]. The overall cellular composition of *N. meningitidis* stain HB-1, grown in minimal medium at a specific growth rate of 0.04 h^-1^ is shown in table S1A.

Table S1A, Overall cellular composition of *N. meningitidis*

|  | *E. Coli*^2^ | N. meningitidis^1^ | |
| --- | --- | --- | --- |
| % (w/w) |  | Average ± SD | RSD assay (%) |
| Protein^3^ | 55 | 71.0 ± 3.6 | 5 |
| RNA^4^ | 20.5 | 8.5 ± 2.2 | 25 |
| DNA | 3.1 | 0.7 ± 0.2 | 15 |
| Lipids^5^ | 9.1 | 11.4 ± 0.6 | 5 |
| LPS^6^ | 3.4 | 5.9 ± 0.3 | 5 |
| Peptidoglycan^7^ | 2.5 | 2.5 ± 0.3 | 10 |
| Glycogen | 2.5 | - | - |
| Polyamines | 0.4 | - | - |
| Metabolites, cofactors, ions | 3.5 | - | - |

^1^ The analytical results were derived from two separate independent chemostat experiments. The standard deviation values (SD) represent the standard deviation between the two experiments and include the relative mean standard deviation (RSD) of the used analytical assay. All macromolecules and their composition were measured unless stated otherwise.

^2^ Average chemical composition of *Escherichia coli* [144].

^3^ Total biomass protein was quantified by summation of the individual measured amino acid concentrations measured in biomass after acid hydrolysis as descibed in the text. The methods recovery was determined as 0.85 ± 4 % based on measurements of pure Bovine Serum Albumin and corrections were made accordingly.

^4^ The measured macromolecules in the biomass accounted for 100 ± 6 % (w/w). The unmeasured content can be explained by a too low measured RNA content. Accurate measurement of total cellular RNA is complicated due to poor stability of RNA. The measured RNA content was multiplied by a factor of 1.2 to correct for unstable RNA [144]. The remainig unmeasured biomass content can account for at least 6.4 % as indicated by the average chemical composition of *E. coli*.

^5^ The fatty acids present in biomass were individually quantified and equally distributed over the different phospholipids using the overall composition of Rahman and co-workers [75] as described in the text. Although the resulting model lipid structures are deviant from the actual stuctures as described by Rahman and co-workers its impact for modeling purposes is negligible.

^6^ LPS was quantified based on the measured amount of 3-hydroxydodecanoic acid (C12:0-3OH), a unique LPS related fatty acid. The LPS value possesses high uncertainty and may need to be readjusted as described in section S1D.

^7^ Peptidoglycan amount in *N. meningitidis* was estimated based on *E. coli* [144] and the composition was based on the average of the peptidoglycan structures present in *N. meningitidis* [101]. For modeling purposes a relative mean standard deviation of 10% was assumed.

The biosynthesis of biomass was set as a linear combination of the macromolecules: protein, DNA, RNA, lipid, peptidoglycan and LPS which were considerd to account for the overall biomass composition. The growth-associated ATP for maintenace was estimated to be 13.27 mol ATP / mol biomass. The accuracy of the estimation is, however, quite low. This value was calculated using linear regression of available 1/Y_x/ATP_ vs growth rate data from *E. coli* [122] using the growth rate applied in this study in the obtained equation followed by conversion to the desired unit using the molecular weight of biomass. One mole of biomass is defined by the reaction equation for this compound (reaction 128 in the simplified model) and is equivalent to 140 g dry weight with an elemental composition given in table S1E. Incorporation of the energy requirement yields the biomass biosynthesis reaction:

#### 0.936 PROT + 0.022 LIPID + 0.003 PEPTIDO + 0.003 LPS + 0.002 DNA + 0.035 RNA + 13.27 ATP + 13.27 H2O > BIOMASS + 13.27 ADP + 13.27 Pi + 13.27 H

#### S1B Protein composition & biosynthesis

Table S1B, Protein composition *N. meningitidis*

|  | N. meningitidis | |
| --- | --- | --- |
| % (mol AA/mol protein) | genome^1^ | Average^2^ ± SD |
| Aspartate (Asp) | 5.3 | 5.1 ± 0.3 |
| Glutamate (Glu) | 6.4 | 7.6 ± 0.4 |
| Serine (Ser) | 5.4 | 4.4 ± 0.2 |
| Histidine (His) | 2.3 | 1.8 ± 0.1 |
| Glycine (Gly) | 6.9 | 9.3 ± 0.5 |
| Threonine (Thr) | 5.2 | 4.7 ± 0.2 |
| Alanine (Ala) | 9.5 | 14.9 ± 0.7 |
| Arginine (Arg) | 5.8 | 5.1 ± 0.3 |
| Tyrosine (Tyr) | 3.2 | 1.9 ± 0.1 |
| Valaline (Val) | 6.8 | 7.2 ± 0.4 |
| Methionine (Met) | 2.6 | 2.6 ± 0.1 |
| Phenylalanine (Phe) | 4.3 | 3.3 ± 0.2 |
| Isoleucine (Ile) | 5.9 | 4.8 ± 0.2 |
| Leucine (Leu) | 10.0 | 7.6 ± 0.4 |
| Lysine (Lys) | 5.8 | 5.4 ± 0.3 |
| Proline (Pro) | 4.2 | 3.5 ± 0.2 |
| Asparagine (Asn)^4^ | 4.1 | 4.0 ± 0.2 |
| Tryptophan (Trp)^3^ | 1.3 | 1.2 ± 0.1 |
| Cysteine (Cys)^3^ | 1.1 | 0.9 ± 0.05 |
| Glutamine (Gln)^4^ | 4.1 | 4.8 ± 0.2 |

^1^ Amino acid composition predicted by the genome [21]. For all genes, a random expression level between 0 - 100000 was used resulting in an identical (random) expression level of the corresponding proteins and their amino acid composition.

^2^ The standard deviation values (SD) represent the standard deviation between two independent chemostat experiments and includes the relative mean standard deviation (RSD) of the used analytical assay.

^3^ The amino acids Cys and Trp (AA) that were destructed during acid hydrolysis were calculated based on the predicted amino acid composition in the genome and the measured threonine quantity by using the formula below.

$$AA=\frac{Thr_{measured}\cdot AA_{genome}}{Thr_{genome}}$$

^4^ The amino acids Gln and Asn (AAn) that were converted to respectively Glu and Asp (AA) during acid hydrolysis were calculated based on the predicted amino acid composition in the genome and the measured Glx and Asx (AAx) quantity by using the formula:

$AAn=\frac{AAx_{measured}\cdot AAn_{genome}}{AA_{genome}}$ in which $AAx_{measured}=\left(AA+AAn\right)_{measured}$

In this way, the ratio of *Glu:Gln* and *Asp:Asn* predicted by the genome is incorporated. This provides a more solid point of departure for flux mass balancing in comparision with the traditional applied 1:1 ratio.

The energy requirement for Protein biosynthesis and processing was assumed identical to

*E. coli*: 4.306 mol ATP / mol protein [145]. Incorporation of this energy requirement yields the final reaction for protein biosynthesis:

1 AA + 4.306 ATP + 3.306 H2O > 1 PROT + 4.306 ADP + 4.306 Pi + 3.551 H

In which 1 AA is the sum of molar fractions of the various amino acids present in *N. meningitidis*.

#### S1C Nucleotide composition & biosynthesis

The DNA composition was derived from the complete nucleotide composition present in the genome sequence [21]. For RNA the Uridine content was based on the Thymine content in the genome sequence [23].

Table S1Ca, DNA composition in *N. meningitidis*

|  | **DNA composition** |
| --- | --- |
|  | % (mol/mol) |
| dTTP | 24.24 |
| dCTP | 25.76 |
| dGTP | 25.76 |
| dATP | 24.24 |
| Molecular weight DNA (g/mol) | 324.96 |

The energy requirement for DNA biosynthesis and processing was assumed identical to

*E. coli*: 1.372 mol ATP / mol DNA [145]. Incorporation of this energy requirement yields the final reaction for DNA biosynthesis:

dNTP + 1.372 ATP + 2.372 H2O > DNA + PPi + 1.372 ADP + 1.372 Pi + 1.372 H

In which dNTP is the sum of molar fractions of the various desoxyribonucleotides present in *N. meningitidis*.

Table S1Cb, RNA composition in *N. meningitidis*

|  | **RNA composition** |
| --- | --- |
|  | % (mol/mol) |
| UTP | 24.27 |
| CTP | 25.56 |
| GTP | 25.97 |
| ATP | 24.20 |
| Molecular weight DNA (g/mol) | 337.63 |

The energy requirement for RNA biosynthesis and processing was assumed identical

*E. coli*: 0.4 mol ATP / mol RNA [145]. Incorporation of this energy requirement yields the final reaction for RNA biosynthesis:

NTP + 0.4 ATP + 1.4 H2O > RNA + PPi + 0.4 ADP + 0.4 Pi + 0.4 H

In which NTP is the sum of molar fractions of the various ribonucleotides present in *N. meningitidis*.

#### S1D Lipid composition & biosynthesis

The fatty acids present in biomass were individually quantified using a modified gas chromatography method as descibed in the text. The quantified fatty acids were equally distributed over the different phospholipids using the overall composition of Rahman and co-workers [75].

Table S1Da, Lipid composition in *N. meningitidis*

|  | *N. gonorrhoeae* | *N. meningitidis* |
| --- | --- | --- |
| % (w/w) | Senff et al.^2^  Average ± SD | Rahman et al.^1^  Average ± SD |
| phosphatidate (PA) | - | 10.2 ± 1.1 |
| phosphatidylethanolamine (PE) | 77 ± 1 | 71.5 ± 4.2 |
| phosphatidylglycerol (PG) | 18 ± 2 | 18.3 ± 4.1 |
| lysophosphatidylethanolamine (LPE) | 2 ± 1 | **-** |
| cardiolipin (CL) | 2 ± 1 | **-** |

^1^ FAB-MS determined average lipid composition of *N. meningitidis* strains NMB, 6940, M7, SS3, CMK2, R6, CMK1 and 469 cultivated in GC broth at 37 °C for 16-18 hours in an aerobic humid atmosphere supplemented with 3.5% CO_2_ [75]. For modeling purposes 10.23 % PA, 71.46 % PE and 18.31 % PG were used.

^2^ Thin layer chromatography (TLC) determined average lipid composition of *N. gonorrhoeae* strain 2686 colonial types 1 and 4 cultivated in Mayer broth at 37 °C for 18 hours in an aerobic humid atmosphere [78]. Earlier, Sud and Feingold [79] determined a comparable composition.

Table S1Db, Fatty acid composition in *N. meningitidis*

| % (mol/mol) | Jantzen et al. ^1^  Average ± SD | Average ± SD^2^ |
| --- | --- | --- |
| dodecanoic acid (C12:0) | 3.5 ± 0.7 | 8.6 ± 0.9 |
| 3-hydroxydodecanoic acid (C12:0-3OH)^3^ | 6.0 ± 2.8 | 9.6 ± 0.2 |
| tetradecanoic acid (C14:0) | 7.0 ± 0.0 | 3.5 ± 0.5 |
| 3-hydroxytetradecanoic acid (C14:0-3OH) | 4.5 ± 2.1 | 4.2 ± 1.1 |
| hexadecenoic acid (C16:1) | 29.0 ± 5.7 | 26.8 ± 1.7 |
| hexadecanoic acid (C16:0) | 39.0 ± 2.8 | 40.6 ± 2.1 |
| octadecenoic acid (C18:1) | 9.0 ± 1.4 | 10.3 ± 0.2 |
| octadecanoic acid (C18:0) | 2.0 ± 0.0 | 1.1 ± 0.1 |
| octadecadienoic acid (C18:2) | 1.0 ± 1.0 | - |

^1^ Gaschromatography (GC) determined fatty acid composition of *N. meningitidis* strains M1 and B8152/66 cultivated on blood agar plates at 37 °C for 20 hours in an aerobic humid atmosphere supplemented with CO_2_ [130].

^2^ GC determined fatty acid composition of *N. meningitidis* strain HB-1 as descibed in the text. The standard deviation values (SD) represent the standard deviation of two independent experiments and does not include the relative mean standard deviation (RSD) of the used analytical assay. Rahman and co-workers [75] additionally identified presence of tetradecenoic acid (C14:1) and hexadecadienoic acid (C16:2) in lipids.

^3^ Based on the measured lipopolysaccharide (LPS) stucture, the molar ratio of C12:0/C12:0-3OH/C14:0-3OH is constant 1/1/1. However, a ratio of approximately 2/2/1 was measured using the GC method. As mentioned in the text, LPS was quantified based on the measured amount of C12:0-3OH (GC), thus neglecting the measured amount of C14:0-3OH. The remaining C12:0 quantity was used in lipid biosynthesis. When the LPS amount is quantified based on the measured amount of C14:0-3OH more C12:0 can be incorporated into lipid but a relatively large amount of C12:0-3OH is left over. Although it is possible that C12:0-3OH is synthesized faster than C14:03OH and accumulates in the cell we assumed that this was unlikely and therfore based the LPS amount on C12:0-3OH. Therefore, the LPS value posseses high uncertainty and may need to be readjusted.

#### S1E Elemental composition

|  | C | H | N | O | P | S | + | - |
| --- | --- | --- | --- | --- | --- | --- | --- | --- |
| Amino Acids |  |  |  |  |  |  |  |  |
| GLU | 5 | 8 | 1 | 4 | 0 | 0 | 0 | 1 |
| GLN | 5 | 9 | 2 | 3 | 0 | 0 | 0 | 1 |
| PRO | 5 | 8 | 1 | 2 | 0 | 0 | 0 | 1 |
| ASP | 4 | 6 | 1 | 4 | 0 | 0 | 0 | 1 |
| ASN | 4 | 7 | 2 | 3 | 0 | 0 | 0 | 1 |
| ARG | 6 | 15 | 4 | 2 | 0 | 0 | 1 | 0 |
| THR | 4 | 8 | 1 | 3 | 0 | 0 | 0 | 1 |
| LYS | 6 | 15 | 2 | 2 | 0 | 0 | 1 | 0 |
| ILE | 6 | 12 | 1 | 2 | 0 | 0 | 0 | 1 |
| MET | 5 | 10 | 1 | 2 | 0 | 1 | 0 | 1 |
| SER | 3 | 6 | 1 | 3 | 0 | 0 | 0 | 1 |
| CYS | 3 | 6 | 1 | 2 | 0 | 1 | 0 | 1 |
| GLY | 2 | 4 | 1 | 2 | 0 | 0 | 0 | 1 |
| VAL | 5 | 10 | 1 | 2 | 0 | 0 | 0 | 1 |
| LEU | 6 | 12 | 1 | 2 | 0 | 0 | 0 | 1 |
| ALA | 3 | 6 | 1 | 2 | 0 | 0 | 0 | 1 |
| HIS | 6 | 10 | 3 | 2 | 0 | 0 | 1 | 0 |
| PHE | 9 | 10 | 1 | 2 | 0 | 0 | 0 | 1 |
| TYR | 9 | 10 | 1 | 3 | 0 | 0 | 0 | 1 |
| TRP | 11 | 11 | 2 | 2 | 0 | 0 | 0 | 1 |
| DNA |  |  |  |  |  |  |  |  |
| dATP | 10 | 12 | 5 | 12 | 3 | 0 | 0 | 4 |
| dCTP | 9 | 12 | 3 | 13 | 3 | 0 | 0 | 4 |
| dGTP | 10 | 12 | 5 | 13 | 3 | 0 | 0 | 4 |
| dTTP | 10 | 13 | 2 | 14 | 3 | 0 | 0 | 4 |
| RNA |  |  |  |  |  |  |  |  |
| ATP | 10 | 12 | 5 | 13 | 3 | 0 | 0 | 4 |
| CTP | 9 | 12 | 3 | 14 | 3 | 0 | 0 | 4 |
| GTP | 10 | 12 | 5 | 14 | 3 | 0 | 0 | 4 |
| UTP | 9 | 11 | 2 | 15 | 3 | 0 | 0 | 4 |
| Fatty Acids |  |  |  |  |  |  |  |  |
| C12:0-3OHACP | 12 | 23 | 0 | 2 | 0 | 0 | 0 | 0 |
| C12:0ACP | 12 | 23 | 0 | 1 | 0 | 0 | 0 | 0 |
| C14:0-3OHACP | 14 | 27 | 0 | 2 | 0 | 0 | 0 | 0 |
| C14:0ACP | 14 | 27 | 0 | 1 | 0 | 0 | 0 | 0 |
| C16:0ACP | 16 | 31 | 0 | 1 | 0 | 0 | 0 | 0 |
| C18:0ACP | 18 | 35 | 0 | 1 | 0 | 0 | 0 | 0 |
| C16:1ACP | 16 | 29 | 0 | 1 | 0 | 0 | 0 | 0 |
| C18:1ACP | 18 | 33 | 0 | 1 | 0 | 0 | 0 | 0 |
| Macromolecules |  |  |  |  |  |  |  |  |
| PA | 35.3 | 65.9 | 0.0 | 8.0 | 1.0 | 0.0 | 0.0 | 0.0 |
| PE | 37.3 | 69.9 | 1.0 | 8.0 | 1.0 | 0.0 | 0.0 | 0.0 |
| PG | 38.3 | 71.9 | 0.0 | 10.0 | 1.0 | 0.0 | 0.0 | 0.0 |
| Peptidoglycan | 55.8 | 88.2 | 12.2 | 27.7 | 0.0 | 0.0 | 0.0 | 1.0 |
| LPS | 134.0 | 245.0 | 4.0 | 67.0 | 3.0 | 0.0 | 0.0 | 4.0 |
| DNA | 9.7 | 12.2 | 3.8 | 7.0 | 1.0 | 0.0 | 0.0 | 2.0 |
| RNA | 9.5 | 11.8 | 3.8 | 8.0 | 1.0 | 0.0 | 0.0 | 2.0 |
| Protein | 4.7 | 7.5 | 1.3 | 1.5 | 0.0 | 0.0 | 0.0 | 0.0 |
| Lipid | 37.3 | 69.8 | 0.7 | 8.4 | 1.0 | 0.0 | 0.0 | 0.0 |
| BIOMASS^1^ | 6.3 | 10.3 | 1.5 | 2.2 | 0.1 | 0.0 | 0.0 | 0.0 |

Based on the measurements an approximate elemental biomass composition was determined:

CH_1.62_O_0.35_N_0.25_P_0.013_S_0.005_. This yields a molecular weight on C-mole basis of ~23 g/C-mole which is comparable with other gram negative organisms like *E. Coli*.

# Metabolites measured in the culture supernatant and off-gas

Table 3, Metabolites measured in the supernatant and off-gas^1^

| Metabolite | Average ± SD^2^ |
| --- | --- |
| Protein^3^ (g.L^-1^) | 0.063 ± 0.019 |
| Asp  Glu  Ser  His  Gly  Thr  Ala  Arg  Tyr  Val  Met  Phe  Ile  Leu  Lys  Pro  Asn  Trp  Cys  Gln | 0.029 ± 0.012  0.020 ± 0.009  0.001 ± 0.001  0.030 ± 0.043  0.004 ± 0.001  0.041 ± 0.012  0.011 ± 0.010  0.012 ± 0.010  0.018 ± 0.007  0.002 ± 0.001  0.002 ± 0.001  0.205 ± 0.058  0.000 ± 0.000  0.007 ± 0.008  0.050 ± 0.020  0.000 ± 0.000  0.000 ± 0.000  0.000 ± 0.000  0.000 ± 0.000  0.000 ± 0.001 |
| lactate | 0.00 ± 0.00 |
| acetate | 0.19 ± 0.04 |
| ethanol | 1.80 ± 0.22 |
| NH_3_ | 4.87 ± 0.97 |
| glucose | 0.00 ± 0.00 |
| off-gas O_2_ %(v/v) | 31.8 ± 4.7 |
| off-gas CO_2_ %(v/v) | 0.25 ± 0.04 |
| k_l_a (h^-1^)^4^ | 13.41 ± 0.56 |

^1^ All metabolites were measured (mM) unless stated otherwise.

^2^ The standard deviation values (SD) represent the standard deviation between two independent experiments and includes the relative mean standard deviation (RSD) of the used analytical assay.

^3^ The compostion of the supernatant protein was assumed to be identical to the cellular protein composition.

^4^ The volumetric oxygen transfer coefficient, k_l_a, in the bioreactor was determined accurately at 37 °C using a steady state set-up and a phosphate buffered saline solution (NVI, Z3000) as reference liquid. As mentioned in the text, the k_l_a values were confirmed using the dynamic method. The determined k_l_a using the dynamic method was 15.73 ± 2.28 h^-1^.
